# Cardiac Magnetic Resonance in Adults: An Updated Review of the Diagnostic Approach to Major Heart Diseases

**DOI:** 10.3390/jcm14207323

**Published:** 2025-10-16

**Authors:** José Ignacio Tudela Martínez, Pablo Alcaraz Pérez, Lourdes Martínez Encarnación, Josefa González-Carrillo, Daniel Rodríguez Sánchez, Francisco Sarabia Tirado, Andrés Francisco Jiménez Sánchez, Florentina Guzmán-Aroca, Juan de Dios Berna Mestre

**Affiliations:** 1 Department of Dermatology, Dentistry, Radiology and Physical Medicine, Faculty of Medicine, University of Murcia, Av. Buenavista, 32, 30120 Murcia, Spain; florentina.guzman@um.es (F.G.-A.); juandeberna@um.es (J.d.D.B.M.); 2 Radiology Department, Virgen De La Arrixaca University Hospital, Ctra. Madrid-Cartagena, s/n, 30120 Murcia, Spain; lourmares@hotmail.com (L.M.E.); danielrs77@hotmail.com (D.R.S.); pasati@hotmail.com (F.S.T.); andresfjs_77@yahoo.es (A.F.J.S.); 3 Radiology Research Group, Biomedical Research Institute of Murcia (IMIB), C/Campo, 12, 30120 Murcia, Spain; 4 Cardiology Department, Virgen De La Arrixaca University Hospital, Ctra. Madrid-Cartagena, s/n, 30120 Murcia, Spain; josegonca.alarcon@gmail.com

**Keywords:** cardiac magnetic resonance, CMR, valvular heart disease, cardiomyopathy, pulmonary hypertension

## Abstract

Cardiac magnetic resonance (CMR) is a non-invasive imaging technique that plays a crucial role in the diagnosis, risk stratification, and management of a broad spectrum of cardiovascular diseases. Its high spatial resolution and ability to provide multiparametric tissue characterization make it uniquely suited for evaluating both structural and functional cardiac abnormalities. This review provides a comprehensive and clinically oriented overview of CMR applications in adult cardiology, structured into six main areas: (1) myocardial scarring in ischemic and non-ischemic cardiomyopathies, (2) infiltrative myocardial diseases, (3) adult congenital heart disease, (4) valvular heart disease, (5) pulmonary hypertension and right ventricular morpho-functional evaluation, and (6) cardio-oncology. In addition, technical considerations are also discussed. Finally, recommendations from recent guidelines issued by main international societies are integrated to support clinical decision-making.

## 1. Introduction

Cardiac (or cardiovascular) magnetic resonance (CMR) has become an essential imaging modality in contemporary cardiology, offering unmatched capabilities in the evaluation of cardiac morphology, function, perfusion, and tissue characterization. Unlike other imaging techniques, CMR provides a comprehensive assessment that combines functional quantification, myocardial characterization, and flow analysis without ionizing radiation.

This review synthesizes guidance from major societies, including the American College of Cardiology (ACC), the American Heart Association (AHA), the European Association of Cardiovascular Imaging (EACVI), the European Society of Cardiology (ESC), and the Society for CMR (SCMR); as well as peer-reviewed literature indexed in PubMed and Scopus up to August 2025. We prioritized guidelines/consensus, metanalyses, and large multicentre studies, complemented by focused primary studies where gaps remained [1,2,3,4,5,6,7,8,9,10]. The content is structured by pathology, with dedicated sections on myocardial scarring (ischemic and not ischemic), infiltrative myocardial diseases, adult congenital heart disease (ACHD), valvular heart disease, pulmonary hypertension (PH), right ventricular (RV) morpho-functional evaluation, and cardio-oncology.

## 2. Myocardial Scarring in Ischemic and Non-Ischemic Cardiomyopathies

Myocardial scars are defined as focal or regional areas of non-viable myocardium, characterized by replacement of normal contractile myocytes with fibrous tissue, most commonly due to infarction [11]. Among the available imaging modalities, late gadolinium enhancement (LGE) with CMR is considered the reference standard for detecting myocardial fibrosis/scarring and assessing viability in both ischemic and non-ischemic cardiomyopathies [9,12,13]. In the setting of myocardial fibrosis, where cell membranes are disrupted and the extracellular matrix is expanded, gadolinium-based contrasts accumulate and lead to regions of hyperenhancement on LGE sequences, indicating irreversible injury [14,15]. The distribution pattern and transmural extent of enhancement enable distinction between ischemic and non-ischemic cardiomyopathies, support assessment of myocardial viability, and provide prognostic insights including the risk of ventricular arrhythmias and sudden cardiac death [11,16].

### 2.1. Ischemic Myocardial Scarring

Ischemic cardiomyopathy, defined by left ventricular (LV) systolic dysfunction due to coronary artery disease (CAD), remains the leading cause of heart failure worldwide [17]. SCMR assigns CMR a Class I recommendation for its diagnostic and prognostic value in both acute and chronic CAD, as well as in myocardial infarction with non-obstructive coronary arteries (MINOCA) [9]. On CMR, ischemic myocardial scars typically follow a coronary distribution and appear as subendocardial or transmural patterns [11]. Cine imaging accurately quantifies ventricular size and function, T2-based sequences identify edema and injured myocardium, and LGE defines scar extent and tissue viability [1,9].

#### 2.1.1. Acute Coronary Syndrome (ACS)

**Figure 1:**
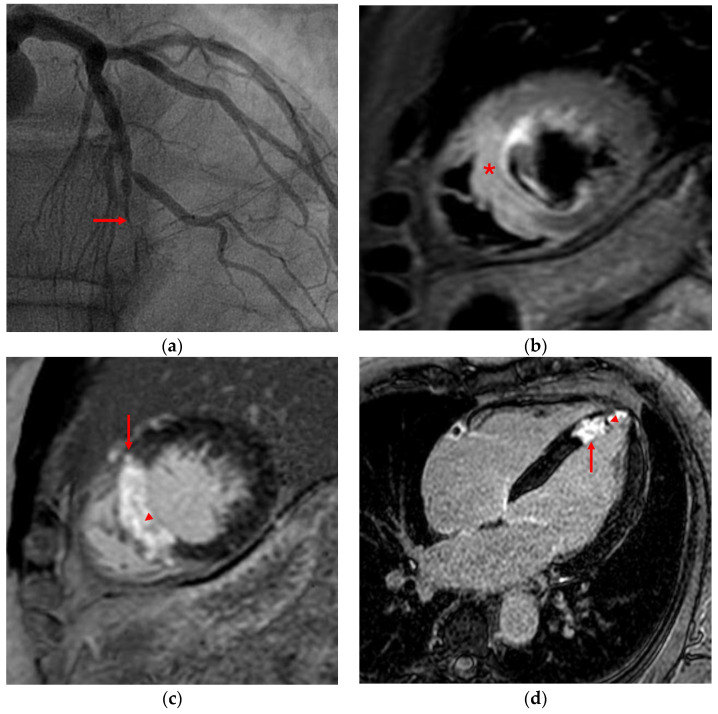
Acute coronary syndrome, five days post-revascularization. (**a**) Coronary angiography: severe two vessel CAD with a complete distal occlusion of left anterior descending (LAD) artery (arrow); (**b**) Short-axis T2 short-tau inversion recovery (STIR) sequence: myocardial edema in the distal LAD territory (*); (**c**) Short-axis LGE sequence: subendocardial scarring (arrow), with MO signs (arrowhead); (**d**) Four-chamber LGE sequence: extensive subendocardial, nearly transmural, ischemic scar in LAD territory (arrow), with MO signs (arrowhead).

In patients with ST-segment elevation myocardial infarction (STEMI), CMR is not indicated prior to reperfusion therapy [1,9]. However, after successful revascularization, CMR provides detailed information on infarct size, microvascular obstruction (MO), myocardial edema, and viability [1,9]. MO appears as a hypointense core within the hyperenhanced infarcted area on LGE images, often surrounded by edema or hemorrhage, and is a strong predictor of adverse remodelling and poor prognosis (Figure 1) [1,9]. According to the ESC guidelines, CMR holds a Class IIa, Level of Evidence (LoE) A recommendation when echocardiographic evaluation is inconclusive, particularly for assessing ventricular structure and function or when LV thrombus is suspected after infarction [1].

In non-ST-segment elevation myocardial infarction (NSTEMI), CMR refines diagnosis and helps guide revascularization strategies in patients without high-risk clinical features [1,9]. Typical findings include subendocardial or patchy scarring in a coronary distribution and, in some cases, MO, which in NSTEMI has been linked to adverse ventricular remodelling and worse clinical outcomes [9,16]. In patients with suspected ACS, but inconclusive biomarkers, absence of ECG changes, and no recurrence of symptoms, stress perfusion CMR offers an alternative to coronary computed tomography angiography (CTA), especially in those with known or extensive CAD, providing incremental diagnostic and prognostic information (Class IIa, LoE A) [1].

#### 2.1.2. Chronic Coronary Syndrome (CCS)

In patients with suspected CCS and a moderate to high pre-test probability of obstructive CAD, functional imaging modalities such as CMR and single-photon emission computed tomography (SPECT) are recommended to estimate the risk of future cardiovascular events (Class I, LoE B) [3,9]. In this context, CMR has demonstrated superior diagnostic performance compared to SPECT, particularly in identifying fixed perfusion defects [9]. Additionally, CMR should also be considered in individuals with suspected CCS and inconclusive echocardiographic examinations (Class IIb, LoE C) [3].

Beyond its diagnostic accuracy, stress CMR has also demonstrated significant cost-effectiveness in patients with suspected or known stable CAD. Randomized and large multicenter trials have shown that stress perfusion CMR not only provides superior accuracy compared with SPECT but also optimizes the use of costly resources such as invasive coronary angiography (ICA) by reducing unnecessary procedures. Moreover, it allows safe discharge of patients with normal results and has proven non-inferior to invasive fractional flow reserve in guiding treatment decisions [9].

#### 2.1.3. MINOCA

Classically, MINOCA is defined as myocardial infarction associated with mild or non-obstructive CAD on ICA [18]. However, MINOCA should be regarded as a diagnostic framework rather than a definitive diagnosis, and its evaluation remains particularly complex [1,6,18,19,20]. It requires a comprehensive integration of clinical, imaging, and laboratory data to identify the underlying cause of myocardial injury in the absence of obstructive CAD. MINOCA encompasses a heterogeneous group of conditions that can be broadly classified into coronary and non-coronary mechanisms. Coronary causes include plaque disruption, spontaneous coronary artery dissection, vasospasm, microvascular dysfunction, and coronary embolism, while non-coronary mechanisms may involve myocardial diseases or systemic processes such as pulmonary embolism or sepsis [1,18]. Once the underlying etiology is determined, the diagnosis should be redefined to direct optimal management strategies and facilitate prognostic stratification [1].

CMR is able to differentiate true MI from non-ischemic myocardial injury, such as myocarditis, primarily through the characteristic pattern of LGE [19]. This diagnostic precision establishes CMR as a pivotal tool in the assessment of patients with suspected MINOCA, holding a Class I, LoE B recommendation in cases where ICA fails to demonstrate an obstructive lesion or an anatomical explanation for the myocardial injury [1,6,9,11].

### 2.2. Non-Ischemic Myocardial Scarring

Non-ischemic myocardial scarring on CMR is defined as fibrotic replacement within the myocardium that occurs independently of CAD. In contrast to ischemic scars, non-ischemic scars typically show atypical distributions with subepicardial or mid-myocardial patterns, and are commonly associated with diverse cardiomyopathies or inflammatory processes. As previously noted, non-ischemic myocardial scarring can be detected during the evaluation of patients with suspected MINOCA [1,18]. Its recognition should lead to reclassification of the clinical entity, according to the specific diagnostic and therapeutic framework of the underlying disease.

#### 2.2.1. Myocarditis and Pericarditis

**Figure 2:**
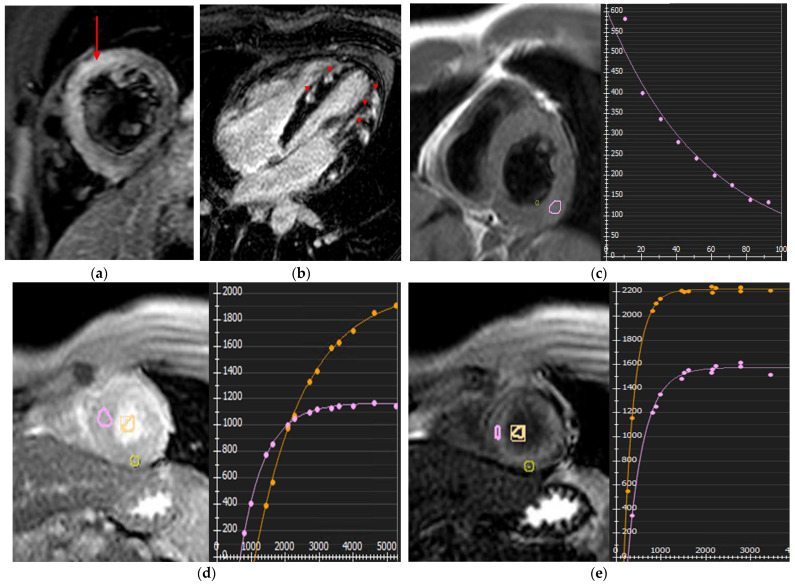
Myocarditis. (**a**) Short-axis T2 STIR sequence: extensive edema in mid-septum and ventricular apex (arrow); (**b**) LGE sequence: non-ischemic subepicardial patchy scarring (arrowheads); (**c**) Quantitative T2 mapping: elevated T2 relaxation time, reaching a maximum of 62 ms in mid lateral wall; (**d**,**e**) Quantitative native (**d**) and post-contrast T1 (**e**) mapping: elevated ECV (31%) in mid/apical lateral and anterior walls.

CMR plays a central role in the evaluation of myocarditis, providing an integrated assessment of ventricular function, myocardial structure, and tissue composition. The updated Lake Louise Criteria (LLC) include the presence of at least one T2-based abnormality in conjunction with one T1-based abnormality for myocarditis diagnosis. T2-weighted imaging (WI) or T2 mapping identifies myocardial edema, which manifests as increased T2 relaxation time, elevated T2 signal intensity (SI), or an increased T2 SI ratio [21,22]. On the other hand, quantitative T1 and extracellular volume (ECV) mapping quantify myocyte injury and interstitial expansion, demonstrated by elevated native T1, increased ECV, or the presence of non-ischemic LGE (Figure 2) [21,22].

CMR holds a Class I (LoE B) recommendation in ESC guidelines, and a Class I (without a formal LoE) in both ACC and SCMR guidelines, for the initial diagnostic approach to myocarditis [9,21,23]. In addition, the most recent ESC guidelines provide a Class I, LoE C recommendation for CMR within 6 months after myocarditis onset, to document resolution or persistence of inflammation, refine risk stratification and therapy, and support return-to-exercise decisions [23].

The ESC guidelines extend this framework to inflammatory myopericardial syndromes, which include pericarditis, myocarditis, and overlapping forms such as myopericarditis or perimyocarditis. In patients with suspected pericardial disease, CMR is recommended when the diagnosis cannot be made using clinical criteria (Class I, LoE B) [23]. In these cases, CMR demonstrates pericardial thickening, edema and LGE, which may reflect ongoing inflammation and neovascularization rather than simple scarring [23]. This capability is particularly relevant in suspected myopericarditis, where CMR can simultaneously demonstrate pericardial and myocardial inflammation. In addition, the SCMR provides a Class I recommendation for CMR in pericardial inflammation and constriction [9].

#### 2.2.2. Cardiomyopathies

Cardiomyopathies are defined as “myocardial disorders in which the heart muscle is structurally and functionally abnormal, in the absence of CAD, hypertension, valvular disease, and congenital heart disease sufficient to cause the observed myocardial abnormality” [24]. The most recent guidelines emphasize a phenotypic classification of cardiomyopathies based on morphological characteristics—such as ventricular dilatation, hypertrophy, and scarring—as well as functional parameters, including both systolic and diastolic dysfunction [2]. In this context, the unique capacity of CMR for detailed myocardial tissue characterization has justified its incorporation into contemporary international cardiology guidelines for the initial diagnostic evaluation of cardiomyopathies (Class I, LoE B) [2,7,9]. Additionally, the ESC guidelines recommend the use of CMR for longitudinal follow-up to assess disease progression in patients with cardiomyopathies (Class IIa, LoE C) [2].

##### Hypertrophic Cardiomyopathy (HCM)

**Figure 3:**
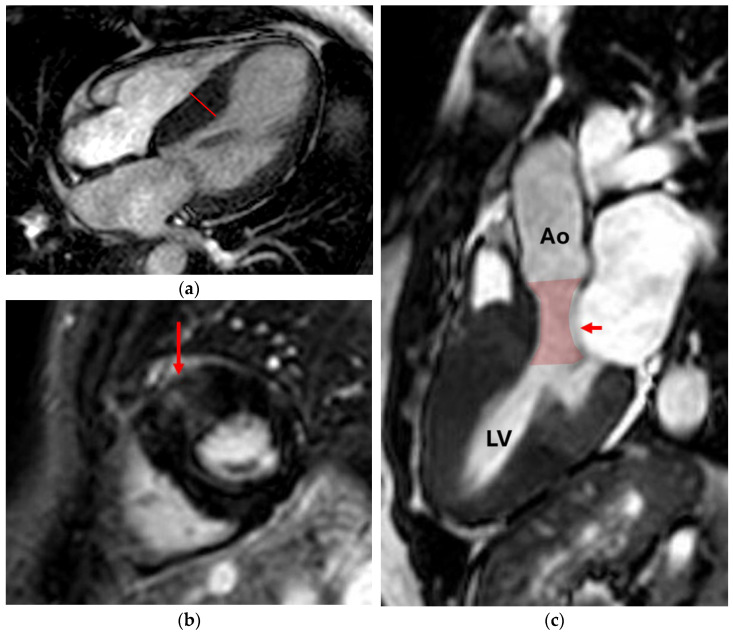
Hypertrophic cardiomyopathy. (**a**) Four-chamber cine sequence: septal hypertrophy with septal thickness of 20 mm (line); (**b**) Short-axis LGE sequence: non-ischemic subepicardial/mesocardiac scarring (arrow); (**c**) Three-chamber cine sequence: left ventricular outflow tract (LVOT) obstruction (highlighted) caused by septal hypertrophy and systolic anterior motion of the mitral leaflets (SAM, arrow), a highly specific finding for HCM [27]. Ao (aorta); LV (left ventricle).

HCM is defined as increased LV wall thickness or myocardial mass that cannot be fully attributed to abnormal loading conditions, and it may occur with or without concomitant RV hypertrophy [24]. In HCM, measurement of myocardial wall thickness can be performed with greater accuracy using CMR than with transthoracic echocardiography (TTE), due to its higher spatial resolution and tomographic capability [25]. Beyond morphologic assessment, CMR facilitates myocardial tissue characterization through LGE, which typically appears as mid-wall punctuate enhancement in a non-coronary distribution (Figure 3). This pattern has been linked to an increased risk of heart failure and sudden cardiac death [24,26,27,28].

CMR also enables comprehensive phenotypic classification of HCM, encompassing asymmetric septal hypertrophy—the most frequent form, accounting for 60–70% of cases—as well as concentric, apical, midventricular, mass-like, and burned-out variants [28]. Cine sequences are employed to assess regional wall motion and chamber dimensions, LGE and T1 mapping sequences are used to detect myocardial fibrosis, and phase-contrast imaging is applied to quantify flow velocities, thereby allowing precise evaluation of LV outflow tract (LVOT) obstruction [9,28].

##### Dilated Cardiomyopathy (DCM)

DCM is characterized by LV dilatation and systolic dysfunction that cannot be fully explained by CAD or abnormal loading conditions, and it is frequently associated with myocardial fibrosis [9,24]. CMR provides superior assessment of LV function, chamber volumes, wall stress, and wall thickening, as well as RV morphology, volumes, and function, when compared with TTE [29,30]. In addition, LGE enables the identification of the characteristic “midwall stripe pattern.” This finding, typically observed as an intramural band of high SI in the basal anteroseptal segment, is present in approximately one-quarter of patients with DCM and has been recognized as a predictor of ventricular arrhythmias and sudden cardiac death [31].

##### Non-Dilated Left Ventricular Myocardiopathy (NDLVC)

The 2023 ESC Guidelines on cardiomyopathies introduced the designation NDLVC, replacing the previous term hypokinetic non-dilated cardiomyopathy. NDLVC is defined by the presence of non-ischemic LV scarring or fatty replacement, with or without associated motion abnormalities, in a non-dilated LV, or by isolated LV dysfunction without evidence of scarring [2]. In most patients, LGE can be detected, generally with a lower mass and extent than in DCM [32]. T1 mapping and ECV quantification provide complementary information on diffuse interstitial fibrosis, while RV ejection fraction (RVEF), left atrial volume index (LAVi), and the presence of epicardial LGE have emerged as important prognostic markers derived from CMR [33].

##### Restrictive Cardiomyopathy (RCM)

RCM is defined as restrictive physiology of the right or left ventricle in the presence of normal myocardial wall thickness and normal or reduced ventricular volumes in both systolic and diastolic phases [24]. It is regarded as the least prevalent form of myocardial disease and may arise from a wide spectrum of etiologies, including infiltrative disorders, which will be discussed separately [33]. CMR can demonstrate atrial enlargement in the setting of preserved ventricular size, exclude pericardial involvement, and provide accurate volumetric assessment using cine sequences [9,33]. Additional techniques contribute to further characterization of RCM: T2WI detects myocardial edema; early gadolinium enhancement allows identification of intraventricular thrombi; and LGE and T1 mapping reveals fibrosis or amyloid infiltration [33]. According to current guidelines, the ESC assigns a Class I, LoE B recommendation for the initial evaluation of RCM and a Class IIa, LoE C recommendation for follow-up, whereas the SCMR provides a Class II recommendation for the use of CMR in this context [2,9].

##### Arrhythmogenic Cardiomyopathy (ACM)

ACM is characterized by progressive fibro-fatty replacement of the myocardium. Although it predominantly involves the RV—a presentation classically referred to as arrhythmogenic RV cardiomyopathy (ARVC)—the recognition of biventricular forms and, less commonly, LV predominant variants has led to the broader adoption of the term ACM, which encompasses the full phenotypic spectrum of the disease [2,34]. The revised Task Force criteria and the more recent Padua criteria incorporate morphological, functional, and structural parameters for the diagnosis of ACM/ARVC. RV abnormalities defined in the updated Task Force criteria include: regional akinesia/dyskinesia or aneurysmal deformation of the free wall, presence of transmural LGE affecting more than one RV region, and histologic evidence of fibrous replacement of the myocardium [35,36]. LV abnormalities, according to the Padua criteria, include global LV systolic dysfunction in the absence of LV dilatation, regional wall hypokinesia/akinesia, and LGE involving more than one segment of the LV free wall [35]. CMR is regarded as the first-line imaging modality for evaluating RV functional and structural abnormalities, having demonstrated superior sensitivity compared with echocardiography [36].

## 3. Infiltrative Myocardial Diseases

While myocardial scarring is a common feature across many non-ischemic cardiomyopathies, infiltrative forms merit a separate discussion because their pathophysiology, imaging features, and clinical implications differ fundamentally. These cardiomyopathies are defined by the deposition of abnormal substances within the myocardium, such as amyloid fibrils or iron. Unlike dilated or hypertrophic cardiomyopathies, where geometric remodelling predominates, infiltrative disease is characterized by profound alterations in tissue composition, best captured by the tissue characterization capabilities of CMR. As with non-infiltrative cardiomyopathies, recent ESC guidelines recommend CMR examination in patients with infiltrative cardiomyopathy at initial evaluation (Class I, LoE B) and suggest its use for follow-up and assessment of therapy response (Class IIa, LoE C) [2]. Similarly, the SCMR recommends performing CMR in infiltrative cardiomyopathies (Class I) [9].

### 3.1. Cardiac Amyloidosis (CA)

**Figure 4:**
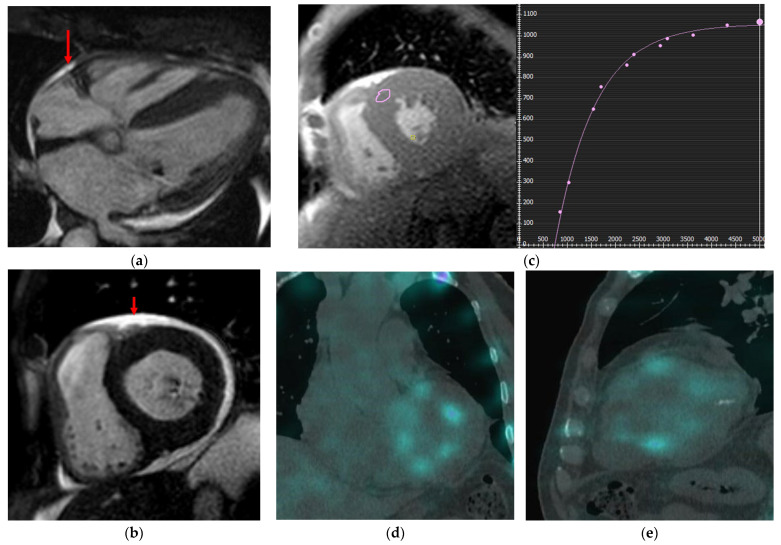
Cardiac amyloidosis. LGE sequences were not acquired due to impaired renal function. (**a**,**b**) Four-chamber (**a**) and short-axis (**b**) cine sequences: diffuse LV wall hypertrophy with left atrial dilatation, and mild pericardial effusion (arrow); (**c**) Native T1 mapping: elevated myocardial T1 values, reaching 1200 ms in the interventricular septum, suggesting amyloid infiltration and diffuse fibrotic changes; (**d**,**e**) coronal (**d**) and sagittal (**e**) reconstructions from Tc_m_^99^-DPD-SPECT: mild and patchy deposit of tracer in myocardium, compatible with amyloid infiltration.

CA is characterized by extracellular deposition of misfolded proteins, most often light-chain (AL) or transthyretin (ATTR) amyloid [37]. This condition is increasingly recognized in patients with heart failure, especially Heart Failure with preserved Ejection Fraction (HFpEF), and is often misdiagnosed as hypertensive or hypertrophic cardiomyopathy, leading to delayed treatment and worse outcomes [38]. CMR is regarded as a highly sensitive and specific tool for CA, enabling morphological assessment, functional analysis, and tissue characterization. Cine imaging may demonstrate concentric or asymmetric ventricular wall thickening, atrial enlargement, and reduced ventricular volumes, while LGE typically shows global subepicardial or transmural patterns with a dark blood pool [38]. T2 mapping may help differentiate AL from ATTR by revealing higher myocardial edema in AL [38]. Additionally, native T1 mapping and ECV quantification are markedly elevated in amyloid infiltration—facilitating the detection of early disease and response to therapy—and are useful for detecting diffuse interstitial fibrotic changes, which can be difficult to assess in LGE sequences [38,39]. These techniques are particularly valuable in patients for whom gadolinium-based contrast agents cannot be administered, a common scenario in the setting of renal involvement associated with systemic amyloidosis (Figure 4).

### 3.2. Anderson-Fabry Disease

Anderson-Fabry disease, or Fabry disease (FD), is an X-linked lysosomal storage disorder caused by deficiency of α-galactosidase A, leading to glycosphingolipid accumulation in multiple organs, including the heart [40]. Cardiac involvement manifests with progressive LV hypertrophy and fibrosis, which represent the main causes of morbidity and mortality due to heart failure and malignant ventricular arrhythmias [41]. CMR plays a central role in early detection, follow-up, and risk stratification. Typical findings include concentric LV hypertrophy, low native T1 values reflecting sphingolipid storage, and LGE that characteristically involves the basal inferolateral wall. Observational data show that the presence and extent of LGE may predict adverse cardiac events, particularly ventricular arrhythmias, highlighting its prognostic value [40]. Additionally, T2 mapping may reveal edema in early inflammatory stages, complementing fibrosis assessment [37].

### 3.3. Cardiac Sarcoidosis

Sarcoidosis is a granulomatous disease with cardiac involvement in up to one-quarter of patients, frequently presenting with arrhythmia, conduction abnormalities, or heart failure [37,41]. Cine CMR detects regional wall motion abnormalities, while T2-weighted sequences identify edema during active inflammation. Most importantly, LGE reveals patchy, often subepicardial or mid-myocardial lesions, commonly involving the basal interventricular septum, ventricular free walls or RV insertion point (“hook sign”) [37,41]. The presence and extent of LGE have prognostic implications, being associated with ventricular arrhythmias and sudden cardiac death [41].

### 3.4. Iron Overload Cardiomyopathy

Iron overload cardiomyopathy may occur in hereditary hemochromatosis, transfusion-dependent anemia, such as β-thalassaemia major or sickle cell disease, or chronic liver disease. CMR T2* mapping is the reference technique for non-invasive quantification of myocardial iron, with values <20 ms indicating clinically relevant overload and <10 ms identifying severe disease associated with adverse outcomes [37]. Native T1 mapping may increase sensitivity, while LGE and ECV reveal fibrosis in advanced disease. These measures allow monitoring of chelation therapy efficacy and prognosis [37,42].

## 4. Valvular Heart Disease

Although TTE is still the preferred and most widely available imaging modality for the evaluation of valvular heart disease, the AHA/ACC, SCMR and ESC guidelines recommend performing CMR when echocardiographic assessment is suboptimal or discrepant with clinical findings [5,9,43,44]. CMR offers added value by combining cine imaging for valve anatomy and function, accurate assessment of great vessels and right-sided structures, and phase-contrast sequences that allow true quantification of regurgitant lesions [39]. In addition, reproducible measurements of ventricular size and function enable comprehensive evaluation of the haemodynamic impact of valve disease and reliable longitudinal follow-up [5,9,44].

### 4.1. Regurgitation

The major advantage of CMR compared to other imaging techniques is its ability to quantify flow, facilitating true calculation of regurgitant valve lesions, which is especially useful in moderate and severe cases, whereas mild regurgitation does not generally require the use CMR [9].

#### 4.1.1. Aortic Regurgitation (AR)

AR may occur because of primary valve disease or aortic root dilatation, with idiopathic degeneration being the most common cause [45]. Its pathophysiology leads to a volume overload of LV, with secondary dilatation and, in advanced cases, aortic dilatation, LV function deterioration, reduced myocardial perfusion and ischemic changes [45]. In patients with moderate or severe AR and suboptimal TTE or discrepancies between clinical and TTE findings, CMR is indicated for LV morpho-functional analysis, aortic size calculation and AR severity estimation (ACC/AHA Class I, LoE B, SCMR Class II) [5,9].

**Figure 5:**
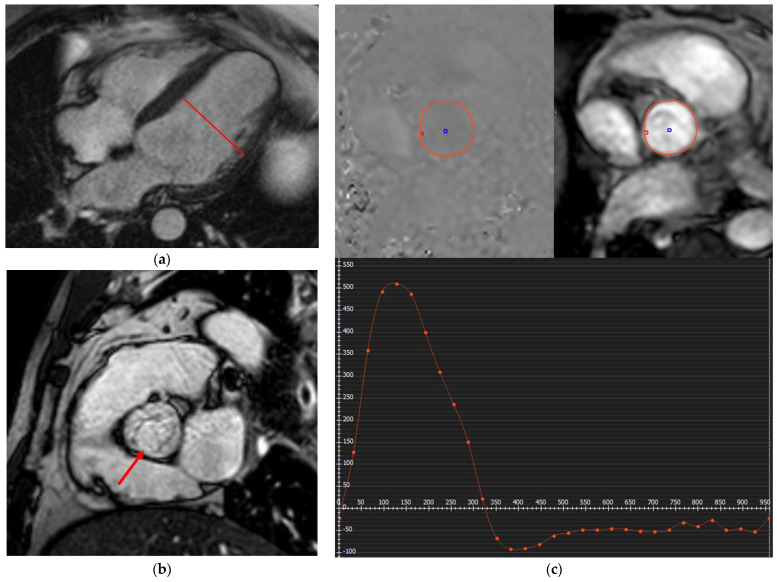
Aortic regurgitation. (**a**) Four-chamber cine view: severe LV dilatation (end-diastolic volume index of 141 mL/m^2^); (**b**) Aortic valve cine sequence: bicuspid aortic valve with incomplete raphe (arrow); (**c**) Quantification of aortic regurgitant volume (highlighted): fraction of 34%.

Generally, AR is graded by regurgitant volume or fraction, which can be quantified by using phase contrast sequences in CMR, demonstrating a better performance than with TTE and associated with clinical worsening [9,46,47]. CMR identifies regurgitant jets, quantifies regurgitant volume and fraction, and enables precise evaluation of LV remodelling and volumes [48]. Additionally, tissue characterization with LGE and mapping techniques reveals focal or diffuse fibrosis, often preceding overt dysfunction, and is associated with adverse remodelling and poorer outcomes (Figure 5) [48].

#### 4.1.2. Mitral Regurgitation (MR)

MR has multiple etiologies, including primary valve disease and secondary causes. In this pathology, left atrial overload results in diastolic volume overload of the LV with consequent dilatation of left cavities [45]. According to AHA/ACC, CMR is indicated to perform morphological and volumetric analysis, as well as MR severity assessment, when there are discrepancies between clinical and echocardiographic findings in cases of primary MR (Class I, LoE B). [5]. In secondary MR, non-invasive imaging (including CMR) is recommended to study MR etiology and myocardial viability (Class I, LoE C) [5]. On the other hand, the SCMR does not differentiate between primary and secondary MR and recommends performing CMR when TTE findings are suboptimal or discrepant (Class II) [9].

CMR provides comprehensive assessment of valve anatomy, accurate quantification of regurgitant volume and fraction, and evaluation of the haemodynamic consequences such as LV dilatation. Advanced sequences like 4D flow further characterize jet morphology, while tissue characterization with LGE and T1 mapping detect myocardial fibrosis, which carries prognostic significance including arrhythmia risk [49,50,51,52].

#### 4.1.3. Tricuspid Regurgitation (TR)

Regarding TR, leaflet morphology can be assessed with contiguous cine images, which is particularly useful in pathologies such as Ebstein’s anomaly. The regurgitant orifice may be measured by planimetry, while quantification is usually performed indirectly by subtracting pulmonary forward flow from RV stroke volume [51]. Additionally, CMR is also the preferred modality for accurate RV volume and function assessment when evaluating secondary regurgitation [43]. The SCMR recommends CMR as Class II for TR [9].

#### 4.1.4. Pulmonary Regurgitation (PR)

In PR, CMR is the reference technique for quantitative assessment of pulmonary regurgitation, although echocardiography is normally preferred in initial approaches. Cine sequences may depict the regurgitant jet, but accurate quantification relies on through-plane velocity mapping positioned just above the valve, which shows good agreement with invasive and non-invasive standards [51]. In addition, CMR provides precise evaluation of RV size and function, which are key determinants for surgical timing [51]. In PR, CMR is especially valuable when acoustic windows are suboptimal or there are discrepancies between imaging and clinical findings (SCMR Class I) [9].

### 4.2. Stenosis

CMR can easily assess any valve stenosis by the use of cine imaging at the valve tips in systole (for aortic and pulmonary stenosis) or diastole (for mitral and tricuspid stenosis), even with irregular outflow tracts [9].

#### 4.2.1. Aortic Stenosis (AS)

In Europe and North America, AS represents the leading native valvular disease requiring surgical or transcatheter treatment, with a rising prevalence driven by population ageing [52,53,54]. AS may occur above, below or within the valve, and the most common cause is idiopathic degeneration of the normal valve [45]. In AS, there is an obstruction of LVOT that results in LV pressure overload and subsequent hypertrophy, which can lead to LV hypoperfusion, ischemia and reduced systolic function [45]. Beyond valve anatomy, CMR provides accurate measurement of LV mass, volumes or strain, and tissue characterization with LGE and T1 mapping detects focal and diffuse fibrosis, both linked to adverse outcomes [48]. Generally, the SCMR recommends performing CMR in AS when echocardiographic assessment is suboptimal or discrepant (Class II), and to identify sub- and supravalvular stenosis (Class I) [9].

#### 4.2.2. Mitral Stenosis (MS)

MS is normally secondary to rheumatic heart disease and involves a left atrial pressure overload, which can lead to PH and RV hypertrophy [45]. MS is generally graded by valve area and transvalvular pressure gradient, which can be accurately visualized by CMR [45,49]. CMR cine imaging demonstrates restrictive valve opening, flow acceleration, and can quantify valve area, while native T1 changes and LGE suggest myocardial fibrosis, which has been linked with post-operative morbidity [49]. However, CMR assessment of MS is still under investigation and recommendations are relatively weak in comparison with other valvopathies (SCMR Class III) [9].

#### 4.2.3. Tricuspid Stenosis (TS)

In TS, CMR can demonstrate restricted leaflets on long-axis views and allows direct planimetry of the valve orifice in diastole, in a similar way to aortic stenosis. This technique shows good agreement with echocardiography, though careful positioning at the valve tips is required for accuracy [51]. Similar to MS, the role of CMR in TS needs further investigation (SCMR Class III) [9].

#### 4.2.4. Pulmonary Stenosis (PS)

In patients with PS, CMR offers excellent visualization of the RV outflow tract and can localize subvalvular, valvular, or supravalvular obstruction. Severity can be assessed qualitatively from valve motion and stenotic jet, or quantitatively by direct planimetry at the valve tips and by measuring peak velocity with phase-contrast imaging. RV mass, function, and associated pulmonary artery stenoses can also be evaluated [51]. The SCMR states CMR as Class I for PS [9].

## 5. Adult Congenital Heart Disease

CMR is central in the evaluation of ACHD, as it integrates anatomic, functional, hemodynamic, and tissue-level assessment within a single examination. Its multiparametric approach includes cine imaging for ventricular volumes and function, phase-contrast sequences for shunt and flow quantification, contrast-enhanced MR angiography for vascular anatomy, and both LGE and quantitative mapping techniques for tissue characterization [8,9,55]. Therefore, international societies consistently position CMR as the reference in ACHD: the AHA/ACC recommends performing serial CMR in ACHD patients at risk of RV enlargement or dysfunction (Class I, LoE B), and in selected complex cases (Class IIa, LoE C); while the SCMR assigns a Class I for ACHD initial evaluation, follow-up, ventricular quantification, and pulmonary-to-systemic flow ratio (Qp:Qs) measurement [8,9]. Since a complete ACHD analysis exceeds the scope of this review, we will highlight the most relevant international recommendations regarding the use of CMR in ACHD.

In shunt lesions, CMR is recommended for a precise delineation of anatomy and measurement of Qp:Qs, with particular value in sinus venosus defects and anomalous pulmonary venous connections, which are challenging to evaluate with TTE (Class I, LoE B) [8,9]. Additionally, the ACC/AHA assigns Class I, LoE B, in cases of atrial septal defects (along with CT and/or TTE) whereas the SCMR assigns a Class II recommendation for these patients. In repaired Tetralogy of Fallot (ToF), CMR is considered the reference standard for quantifying RV size, RV function, and pulmonary regurgitation, which are key parameters guiding the timing of pulmonary valve replacement (Class I, LoE B) [8,9,55]. In d-transposition of the great arteries (dTGA), CMR is recommended after an arterial switch to evaluate the systemic RV, detect myocardial fibrosis, and assess for baffle obstruction or leaks (Class I, LoE C) [8,9]. Additionally, annual imaging examination with CMR or echocardiography is recommended to provide surveillance of the neoaortic root, semilunar valves, pulmonary arteries, and coronary arteries (Class I, LoE C) [8]. In congenitally corrected TGA (ccTGA), CMR is recommended to determine systemic RV dimensions and systolic function (SCMR Class I; ACC/AHA Class IIa, LoE C) [8,9]. In coarctation of the aorta, CMR and/or CTA are recommended before and after intervention, providing information on the site and severity of narrowing, collateral circulation, and associated vascular changes (Class I, LoE B) [8,9,55]. Finally, in single ventricle circulation and Fontan palliation, annual imaging examination with either CMR or echocardiography is recommended for assessment of ventricular volumes, systemic-to-pulmonary collaterals, conduit and baffle patency, and thrombus detection (Class I, LoE C) [8,9,55].

Taken together, across different scenarios of ACHD, CMR provides a comprehensive, one-stop-shop approach that combines anatomical visualization, reference-standard quantification of ventricular volumes and function, shunt and flow measurements, tissue characterization, and vascular imaging.

## 6. Pulmonary Hypertension and RV Morpho-Functional Evaluation

### 6.1. Pulmonary Hypertension

PH is defined as a mean pulmonary artery (PA) pressure >20 mmHg on catheterization. It has a multifactorial etiology, including pulmonary arterial hypertension, PH due to left heart disease, PH associated with lung disease and/or hypoxia, chronic thrombo-embolic PH, and PH with unclear or multifactorial mechanisms [56,57]. Current ESC guidelines have incorporated CMR-derived metrics into risk stratification algorithms for PH, and assign a Class IIb, LoE C, recommendation for its use in symptomatic systemic sclerosis patients, to guide the decision for catheterization [57]. Similarly, the American College of Radiology (ACR) supports the use of CMR for assessment of RV morphology and function in PH suspected patients [10]. However, no formal Class of Recommendation or LoE has yet been assigned for patients with suspected PH, underlining the need for further studies [57].

CMR provides both diagnostic and prognostic insights in PH affected patients, with key findings including: (1) RV dilation, hypertrophy and reduced RVEF <45%; (2) systolic septal flattening (“D-shaped” LV), (3) main PA dilation, (4) reduced PA relative area change between systole and diastole <15%, which predicts poor prognosis, and (5) LGE at RV insertion points, which reflects chronic pressure overload [58,59,60,61,62]. Additionally, CMR-derived RVEF and RV end-systolic volume index (RVESVi) have been proposed as robust predictors of mortality and clinical worsening, as demonstrated in a previous meta-analysis [60]. Finally, Novel 4D flow CMR allows evaluation of vortical flow, energetics, and PA hemodynamic, increasing the diagnostic and prognostic utility of CMR in PH-affected patients [60,62,63,64].

### 6.2. RV Morpho-Functional Evaluation

The complex morphology and retrosternal location of RV make it difficult to assess by echocardiography and TTE. As a result, international guidelines consistently identify CMR as the reference standard for quantitative assessment of RV morphological and functional metrics [8,9,63,64]. The EACVI, as well as the ESC, SCMR and AHA/ACC consider performing CMR in cases where accurate and reproducible quantification of RV is required, such is congenital heart disease, ARVC or when echocardiography is inadequate (Class I, LoE B) [8,9,57,65,66].

## 7. CMR in Cardio-Oncology

Cardiac masses represent a frequent diagnostic challenge in oncologic patients. According to the SCMR consensus document, CMR holds a Class I indication for the evaluation of suspected cardiac masses, differentiation of tumor versus thrombus, distinction between benign and malignant lesions, guiding surgery or biopsy, follow-up of benign tumors, evaluation after resection or in suspected recurrence/progression post-chemotherapy or radiotherapy, assessment of extracardiac extension, and appraisal of hemodynamic consequences. Its multiparametric approach with cine, perfusion, and tissue characterization sequences establishes CMR as the reference method for comprehensive assessment of cardiac masses (SCMR Class I) [9]. The ESC Cardio-Oncology Guidelines also include CMR as a central modality in the diagnostic algorithm of cardiac masses, particularly when echocardiographic findings are inconclusive or tissue characterization is required, although no formal Class of Recommendation is specified [67].

Beyond cardiac masses, cancer therapies can induce a wide range of cardiovascular complications, including LV dysfunction, myocarditis, ischemia, and pericardial or valvular disease. According to ESC Guidelines, CMR should be considered for the assessment of cardiac function when TTE is unavailable or not diagnostic (Class I, LoE B). Additionally, CMR is recommended in suspected myocarditis after cancer therapy (Class I, LoE B), as well as in immune checkpoint inhibitor (ICI) related myocarditis or pericarditis (Class I, LoE C). Finally, in patients with cancer therapy–related LV dysfunction or myocarditis, CMR should be considered during follow-up to monitor resolution or persistence of inflammation and remodelling (Class IIa, LoE C) [67].

## 8. Discussion

CMR has evolved into an indispensable tool in adult cardiology, uniquely integrating structural, functional, and tissue-level assessment within a single examination. It is now the reference standard for RV evaluation, myocardial fibrosis detection, ACHD, and multiparametric phenotyping of cardiomyopathies, and it also delivers added diagnostic and prognostic value in valvular disease, infiltrative disorders, PH, and cardio-oncology.

Taken together, across the different cardiac conditions reviewed, CMR has been consistently incorporated into major international guidelines (Table 1 and Table 2). According to ACC/AHA and ESC guidelines, CMR recommendations are classified into three categories: Class I (evidence/general agreement that CMR is useful); Class II (conflicting evidence or divergence of opinion regarding usefulness), with subclass IIa (evidence favors usefulness) and IIb (usefulness is not well established); and Class III (evidence/general agreement that CMR is not useful and may be harmful). Levels of evidence are defined as A (data from multiple randomized clinical trials or meta-analyses), B (data from a single randomized clinical trial or large non-randomized studies), and C (expert consensus and/or small studies, retrospective studies or registries) [44].

In contrast, the SCMR uses a distinct three-class system, based on clinical utility (Table 2): Class I (appropriate, well supported); Class II (useful, limited evidence); and Class III (useful, but rarely required). This system does not provide a formal LoE.

To complement the guideline summary in Table 1 and Table 2, Table 3 presents a focused and summarized comparison of echocardiography, CMR, and CT/SPECT imaging. Modality selection should be individualized based on the clinical scenario, pre-test probability, availability, contraindications, and radiation considerations.

Although CMR is now considered a central technique in the assessment of major heart diseases, several gaps in knowledge remain. Regarding ACHD, both AHA/ACC and ESC guidelines emphasize the challenge of generating evidence in these heterogeneous, low-prevalence populations, underscoring the need for multicenter registries, innovative study designs, and structured care networks to prevent loss to follow-up [8,55]. In valvular heart disease, the role of CMR in mitral and tricuspid stenosis is limited, reflected by only weak or absent guideline recommendations (SCMR Class III) [9]. Besides, in PH, while CMR-derived metrics have shown prognostic value, guideline endorsement is currently restricted to selected subgroups, such as symptomatic patients with systemic sclerosis, without a formal Class of Recommendation and LoE for general patients [8]. Furthermore, quantitative mapping lacks universal prognostic cut-offs since native T1/T2 values are system- and sequence-dependent. In this context, the SCMR/EACVI recommend site-specific reference ranges, advise against reporting native T1/T2 clinically in the absence of local ranges, and indicate that results should be compared only when acquired under similar conditions [68]. Finally, the evidence regarding CMR cost-effectiveness remains sparse and concentrated in CAD pathway’s, limiting firm conclusions in other clinical scenarios [9].

Beyond current CMR applications, artificial intelligence (AI) is driving a paradigm shift that extends well beyond post-processing. Deep learning–based acquisition and reconstruction protocols have already demonstrated the ability to reduce scan times, streamline protocols, and improve patient comfort without loss of diagnostic accuracy [69,70]. At the same time, AI pipelines are also advancing toward standardized, quantitative, and automated reporting by enabling automated segmentation, flow quantification, and tissue characterization across centers, thereby improving reproducibility [70,71]. However, many of these tools remain embedded in research platforms and are not fully integrated into clinical reporting systems, which limits their widespread use in daily practice. Bridging this gap will be essential for routine adoption [71].

## 9. Conclusions

CMR is an established imaging modality in adult cardiology, endorsed by international guidelines for RV assessment, myocardial fibrosis, ACHD, and cardiomyopathies, with additional value in valvular disease, and cardio-oncology. However, important knowledge gaps persist, including limited evidence in ACHD due to small and heterogeneous cohorts, incomplete translation of prognostic markers in PH into recommendations, lack of standardized cut-offs for quantitative mapping, and scarce cost-effectiveness data. AI is already reshaping CMR, with deep learning–based acquisition and reconstruction reducing scan times and improving patient experience, while automated pipelines for segmentation, flow quantification, tissue characterization, and reporting enhance standardization and reproducibility. Widespread adoption will require its incorporation into clinical workflows and guidelines, allowing CMR to evolve into a faster, quantitative, and automated modality in routine practice.

## Figures and Tables

**Table 1:** Main recommendations for CMR examination in different scientific societies.

Indication for CMR	Class	LoE	Society
Acute coronary syndrome ^1^	IIa	A	ESC
Chronic coronary syndrome (moderate-high probability of obstructive CAD)	I	B	ESC
Chronic coronary syndrome (inconclusive echocardiographic examination)	IIb	C	ESC
MINOCA	I	B	ACC/AHA, ESC
Myocarditis (general, cancer therapy-related)	I	B	ACC/AHA, ESC
Myocarditis (follow up, ICI-related)	I	C	ESC
Pericarditis (undiagnosed with clinical criteria)	I	B	ESC
Cardiomyopathies ^2^ (initial assessment)	I	B	AHA/ACC, ESC
Cardiomyopathies ^2^ (follow-up)	IIa	C	ESC
Aortic regurgitation ^3^	I	ACC/AHA	B
Mitral regurgitation (primary) ^3^	I	B	ACC/AHA
Mitral regurgitation (secondary) ^3^	I	C	ACC/AHA
Adult congenital heart disease (shunt lesions ^4^, ToF, coarctation of aorta)	I	B	ACC/AHA
Adult congenital heart disease (dTGA, single ventricle and Fontan circulation)	I	C	ACC/AHA
Pulmonary hypertension (symptomatic systemic sclerosis patients)	IIb	C	ESC
Right ventricular morpho-functional evaluation	I	B	ACC/AHA, EACVI/ESC
Cancer therapy-related LV dysfunction follow-up	IIa	C	ESC

^1^ After reperfusion treatment. ^2^ Including infiltrative cardiomyopathies. ^3^ In valvular heart disease, CMR is indicated when echocardiographic assessment is inconclusive or there are discrepancies between clinical and echocardiographic findings. ^4^ Partial anomalous pulmonary venous connections and atrial septal defects.

**Table 2:** Main recommendations for CMR examination according to the SCMR.

Indication for CMR	Class
Coronary artery disease	I
MINOCA	I
Myocarditis	I
Pericardial inflammation and constriction	I
Cardiomyopathies ^1^	I
Aortic, mitral and tricuspid regurgitation ^2^	II
Pulmonary regurgitation and stenosis ^2^	I
Aortic stenosis (general) ^2^	II
Aortic stenosis (sub- and supravalvular stenosis) ^2^	I
Mitral and tricuspid stenosis ^2^	III
Adult congenital heart disease ^3^	I
Right ventricular morpho-functional evaluation	I
Cardiac mass assessment	I

^1^ Including infiltrative cardiomyopathies. ^2^ In valvular heart disease, CMR is indicated when echocardiographic assessment is inconclusive or there are discrepancies between clinical and echocardiographic findings. ^3^ Including: general approach, anomalous pulmonary venous connection, sinus venosus defects, ToF, dTGA, ccTGA, and single ventricle heart disease.

**Table 3:** Comparison of main imaging modalities in different clinical tasks.

Clinical Task	Echocardiography	CMR	CT/Nuclear Imaging
Morphology, function and valvular evaluation	First line for morphology and LV/RV function; may underestimate volumes and regurgitant severity	Reference standard for ventricular volumes, mass, and flow quantification	CT offers excellent valvular anatomy, but limited hemodynamics
Tissue characterization and myocardial scarring	Indirect signs only (wall thickening, strain changes)	LGE and quantitative mapping detect scar, edema, and infiltration	Nuclear scans show perfusion and metabolism
Vascular anatomy	Limited use	MR angiography (selected cases)	CTA is the reference for noninvasive coronary imaging
Ischemia detection	Stress echo provides functional ischemia testing	Stress perfusion CMR is highly accurate and cost-effective	SPECT is widely available, but less accurate
Radiation/contrast	No radiation and widely available, but operator-dependent	No radiation, but requires gadolinium use and longer exam times	Involves ionizing radiation and uses iodinated contrast (CT) or tracers (SPECT)

## Data Availability

No new data were created or analyzed in this study.

## Abbreviations

The following abbreviations are used in this manuscript:

ACHD	Adult congenital heart disease
ACS	Acute Coronary Syndrome
ACM	Arrhythmogenic Cardiomyopathy
AR	Aortic Regurgitation
ARVC	Arrhythmogenic Right Ventricular Cardiomyopathy
AS	Aortic Stenosis
CAD	Coronary Artery Disease
CCS	Chronic Coronary Syndrome
CMR	Cardiac Magnetic Resonance
DCM	Dilated Cardiomyopathy
ESC	European Society of Cardiology
HCM	Hypertrophic Cardiomyopathy
LGE	Late Gadolinium Enhancement
LoE	Level of Evidence
LV	Left Ventricle
MINOCA	Myocardial Infarction with Non-Obstructive Coronary Arteries
MR	Mitral Regurgitation
MS	Mitral Stenosis
NDLVC	Non-Dilated Left Ventricular Cardiomyopathy
PH	Pulmonary Hypertension
PR	Pulmonary Regurgitation
PS	Pulmonary Stenosis
RCM	Restrictive Cardiomyopathy
RV	Right Ventricle
TR	Tricuspid Regurgitation
TS	Tricuspid Stenosis
TTE	Transthoracic Echocardiography
